# Effects of Heating, Pelleting, and Feed Matrix on Apparent Concentrations of Cereal Ergot Alkaloids in Relation to Growth Performance and Welfare Parameters of Backgrounding Beef Steers

**DOI:** 10.3390/toxins14090580

**Published:** 2022-08-24

**Authors:** Kim Stanford, Karen S. Schwartzkopf-Genswein, Daniela M. Meléndez, Skyler Ngo, Michael Harding, Tim A. McAllister, Dian Schatzmayr, Mary Lou Swift, Barry Blakley, Gabriel O. Ribeiro

**Affiliations:** 1 Department of Biological Sciences, University of Lethbridge, 4401 University Dr. Lethbridge, Lethbridge, AB T1K 3M4, Canada; 2 Agriculture and Agri-Food Canada Research and Development Center, 5401-1st Ave. S. Lethbridge, Lethbridge, AB T1J 4B1, Canada; 3 Alberta Agriculture, Forestry and Rural Economic Development, Crop Diversification Center South, Brooks, AB T1R 1E6, Canada; 4 DSM–BIOMIN Research Center, Technopark 1, 3430 Tulln, Austria; 5 Department of Agricultural, Food and Nutritional Sciences, University of Alberta, Edmonton, AB T6G 2P5, Canada; 6 Department of Veterinary Biomedical Sciences, University of Saskatchewan, Saskatoon, SK S7N 5A2, Canada; 7 Department of Animal and Poultry Science, University of Saskatchewan, Saskatoon, SK S7N 5A8, Canada

**Keywords:** ergot, ergot alkaloids, cattle, feed processing, pellet, welfare, feed mycotoxins, emerging mycotoxins

## Abstract

As the contamination of cereal grains with ergot has been increasing in Western Canada, studies were undertaken to evaluate the impacts of heating (60, 80, 120, or 190 °C) alone or in combination with pelleting on concentrations of ergot alkaloids. Fifteen samples of ergot-contaminated grain from Alberta and Saskatchewan were assayed for *R* and *S* epimers of six alkaloids (ergocryptine, ergocristine, ergocornine, ergometrine, ergosine, and ergotamine) using HPLC MS/MS. Five samples with distinct alkaloid profiles were then selected for heating and pelleting studies. Heating resulted in a linear increase (*p* < 0.05) of total *R* and total *S* epimers with increasing temperature, although some individual *R* epimers were stable (ergometrine, ergosine, ergotamine). Pelleting also increased (*p* < 0.05) concentrations of total *R* and total *S* epimers detected, although ergometrine concentration decreased (*p* < 0.05) after pelleting. A feeding study arranged in a 2 × 2 factorial structure used 48 backgrounding Angus-cross steers fed four different diets: (1) Control Mash (CM, no added ergot), (2) Control Pellet (CP), (3) Ergot Mash (EM), or (4) Ergot Pellet (EP). Pelleting heated the ergot to 90–100 °C under 4 bars pressure, but the ergot used in the feeding study was not otherwise heated. Alkaloid concentrations of EM and EP varied by up to 1.1 mg/kg depending on the feed matrix assayed. No differences among treatments were noted for growth performance, feed intake, feed conversion, concentrations of serum prolactin and haptoglobin, hair cortisol, or in temperatures of extremities measured by infrared thermography. The only negative impacts of ergot alkaloids were on blood parameters indicative of reduced immune function or chronic inflammation. Pelleting did not heighten the negative clinical outcomes of ergot, although alkaloid concentrations of pelleted feed increased depending on the matrix assayed. It was hypothesized that the heat and pressure associated with pelleting may enhance the recovery of alkaloids from pelleted feed.

## 1. Introduction

As the incidence of contamination of cereal crops with the fungus *Claviceps purpurea* has increased in Canada over the past 25 years [1,2], economical methods are required to allow contaminated grain to be used as livestock feed while avoiding the negative impacts of toxic ergot alkaloids as part of an integrated mycotoxin management system [3]. Allowable concentrations of ergot alkaloids in diets for beef cattle in Canada are currently 2 to 3 mg/kg although these limits are under review, with a 1 mg/kg limit proposed [4]. In the past, it was common to assess only *R* alkaloid epimers, as *S* epimers were thought to be biologically inert [5,6]. However, work by Cherewyk et al. [7,8] demonstrated the vasoconstrictive potential of cereal alkaloid *S* epimers, reinforcing the need for their evaluation. Even when *R* and *S* epimers are assayed, evaluating the potential toxicity of ergot is difficult, as interactions occur among alkaloids, and their net effect cannot be predicted [9]. Accordingly, a better understanding is required of the concentrations of ergot alkaloids and their epimers, which produce negative physiological effects, a point defined as the ‘lowest observed adverse effect level’ (LOAEL) [10]. Ergot alkaloids are thought to negatively impact the growth performance of livestock by reducing feed intake and/or by interfering with the activity of hormones, such as norepinephrine, dopamine, and serotonin [11]. Other negative physiological impacts of ergot alkaloids include lameness, gangrene in extremities, complete cessation of milk production, abortions, and death [5].

Grain processing techniques, such as heating and pelleting, have been evaluated as potential strategies for reducing concentrations of cereal ergot alkaloids. Merkel et al. [12] reported the degradation of alkaloids during baking of contaminated rye flour at 190 °C and a shift from *R* to *S* epimers for all alkaloids. Similarly, exposure to both steam (100 °C) and 40 bar mechanical pressure caused an 11% reduction in total ergot alkaloids and a shift toward *S* epimers [10]. Tittlemier et al. [13] cooked contaminated pasta at 100 °C, and while no overall losses of ergot alkaloids were detected, a shift toward *S* epimers occurred. A previous study by our laboratory determined that pelleting feed, which exposed ergot to 90–100 °C and 4 bar pressure, significantly increased average daily gain and serum prolactin concentrations in lambs compared to those fed mash diets with a similar concentration of *R* epimers (433 µg/kg) [14]. However, our previous study was based on a single ergot alkaloid profile, and *S* epimers were not evaluated. Accordingly, the present study was undertaken to evaluate the effects of heating (60, 80, 120, or 190 °C) alone or in combination with pelleting on alkaloid profiles in multiple samples of ergot-contaminated grain. Based on changes in *R* and *S* epimers, a feed-processing treatment was then selected for use in a feeding study. Backgrounding cattle newly arrived to the feedlot were used and fed diets containing 60% forage, comparing growth performance and welfare measures to those of cattle receiving either unprocessed ergot, or diets with no added ergot. Welfare measures included hair cortisol as a measurement of long-term stress [15], serum haptoglobin as a marker for inflammation [16], and infrared thermography of extremities to measure changes in temperature related to vasoconstriction after exposure to ergot alkaloids [17].

## 2. Results

### 2.1. Sources of Ergot and Impacts of Heating/Pelleting

Sources of ergot used in heating and pelleting studies differed (*p* < 0.05) in concentrations of all *R* and *S* epimers as well as in total alkaloids (Table 1), although ratios of epimers were more consistent. The ratio of ergotamine with its *S* epimer did not differ among sources, and four of five samples had similar ratios of ergocornine and ergocristine respective to *S* epimers, with only sample 24 differing (*p* < 0.05).

Heating the ergot resulted in a linear increase (*p* < 0.05) in the detection of both total *R* and total *S* epimers with increasing temperature (Table 2). However, some individual *R* epimers did not change in concentration with heating (ergometrine, ergosine, and ergotamine). Quadratic effects of heating on alkaloid concentration were noted for total and all individual *S* epimers, but of *R* epimers only ergocryptine showed a quadratic increase (*p* < 0.05) with heating. Similarly, pelleting also resulted in an increase (*p* < 0.05) in concentrations of total *R* and total *S* epimers (Table 3), although among individual alkaloids, ergometrine decreased (*p* < 0.05) after pelleting, while ergocristine, ergocristinine, and ergotaminine increased (*p* < 0.05). In contrast to heating, ratios of *R* to *S* epimers remained stable after pelleting, with only ergometrine: ergotmetrinine being reduced (*p* < 0.05). With heating, ratios of *R* to *S* epimers showed linear and quadratic reductions (*p* < 0.05) with increased temperature; with ergocryptine:ergocryptinine and ergocornine:ergocorninine being the exceptions, which only showed quadratic reductions.

Evaluating combinations of heat and pelleting, unheated ergot in mash form had the lowest (*p* < 0.05) concentration of total alkaloids compared to other treatments, although there were some exceptions among individual alkaloids (Table 4). Concentrations of ergometrinine were lowest (*p* < 0.05) in ergot that was unheated prior to pelleting, while those of ergometrine were lowest (*p* < 0.05) in ergot that was heated to 120 °C prior to pelleting. However, the greatest concentrations (*p <* 0.05) of *S* epimers were generally found in ergot that had been pelleted after heating to 190 °C. Ergocryptine:ergocryptinine was not affected by a combination heating and pelleting, but also tended (*p* = 0.07) to be lowest for ergot heated to 190 °C before pelleting. As the lowest total alkaloid concentrations were found in ergot, which was not subject to any processing, and heating, except for that occurring during pelleting, can be avoided, the cattle-feeding study compared unheated ergot in mash form (EM) to pelleted ergot (EP), which had not been otherwise heated prior to pelleting.

### 2.2. Ergot Alkaloid Analyses of Supplements and Complete Diets

Only trace amounts of alkaloids were detected in the control diets without added ergot (Table 5). A concentration of 1.75 mg/kg total ergot alkaloids was targeted for diets containing added ergot, and this was achieved for the EP diet, although the total alkaloids detected in the EM diet were 55% higher than those of EP. However, when only the supplement portion of the diet was evaluated, the alkaloid concentrations detected in EM were 39% lower than those of the EP supplement. These changes were related to reduced *S* epimers detected in the EM supplement (28% of those detected in the EM diet) and increased *R* epimers in the EP supplement (167% of those detected in the EP diet). As the complete diets contained silage, their pH was 4.7, while that of the supplements was 5.6. The dry matter content of complete diets averaged 54.6% (Table 6), as silage was 60% of the diet on a DM basis, while the DM content of supplements averaged 94% (data not shown).

### 2.3. Growth Performance of Cattle and Physiological Impacts

Initial weight of cattle, average daily gain (ADG), feed intake, or gain:feed did not differ (*p* > 0.44) among treatments (Table 7). Similarly, serum prolactin levels did not differ (*p* = 0.84; Table 8) among the treatment groups. However, an interaction (*p* = 0.04) of ergot by diet form was found for hair cortisol, with EP cattle showing higher hair cortisol than CP-fed cattle. Similar to rectal temperature, which did not vary across treatments, infrared thermography of the ear, coronary band, and tail head showed no treatment differences. In contrast to other physiological measurements, the CBC results showed the impacts of feeding ergot (Table 8). Cattle-receiving diets containing ergot had lower counts of total white blood cells (*p* = 0.02), and an ergot by diet form interaction revealed that this reduction was most evident for EP-fed cattle (*p* = 0.03). Of the white blood cells, both monocytes and granulocytes were reduced (*p* = 0.01) in the ergot-fed cattle, while lymphocyte concentrations were not affected (*p* = 0.52). As a percentage, lymphocytes were increased (*p* = 0.001) in ergot-fed cattle, as the percentages of both monocytes and granulocytes were reduced (*p* = 0.001). The concentration of hemoglobin was reduced (*p* = 0.001) in cattle fed ergot, and again this was most pronounced (*p* = 0.05) in the EP group, although % hematocrit was not affected by ergot (*p* = 0.14), as total red blood cell counts were higher (*p* = 0.04) in cattle fed ergot.

Nutrient analyses of orts showed no signs of sorting of ergot-containing supplements by cattle (Table 6). However, cattle receiving CM may have preferentially consumed the silage component of the diet due to the heightened crude protein and ash, and reduced acid detergent fiber and neutral detergent fiber in orts (indicative of supplement refusals) as compared to those from cattle receiving other treatments.

## 3. Discussion

### 3.1. Sources of Ergot and Impacts of Heating/Pelleting

Increasing concentrations of *S* epimers as a result of heating cereal ergot has been previously noted [10,12,13], but in the present study, total *R* epimers also increased with increasing temperature. In some studies, reductions in *R* epimers outweighed increases in *S* epimers, and total alkaloids decreased after heating [10,12]. Ergotamine and ergosine, two of the three alkaloids that did not change in concentration with heating, have previously shown stability when heated to 190 °C [12]. Other studies [13] did not find a decrease in total alkaloids after heating, a response that is likely dependent on the type and proportions of alkaloids in the ergot, temperature, and whether dry or moist heat is applied. Most studies [10,12,13] have evaluated a single source or blended ergot instead of distinct sources. Similar to the present study, Schummer et al. [18] heated multiple sources of ergot to 100 °C and noted a decrease in total alkaloids in only certain samples, which was attributed to a lack of homogeneity even after careful blending and grinding of the ergot-contaminated grain. In the present study, a lack of homogeneity may have led to sporadic increases in alkaloid concentrations after heating, but this would not explain the linear increases with temperature for most alkaloids. Increased alkaloids after heat treatment were noted by Tittlemier et al. [13] and Danicke [10], but were also attributed to heterogeneous concentrations of alkaloids influencing alkaloid concentrations after heating and re-sampling.

Although a few studies [10,13,18] have evaluated the impact of heating on the concentration of ergot alkaloids and epimerization, the effects of pelleting have been less studied. Our previous study [14] measured only *R* epimers and speculated that changes in epimerization might have been responsible for the increased growth performance of lambs fed diets containing pelleted ergot. As the present study showed increased detection of *R* and *S* epimers after pelleting, this previous hypothesis is unlikely. Additionally, pelleted and mash diets in the study by Coufal-Majewski et al. [14] were balanced to contain equal concentrations of ergot alkaloids. Less ergot-contaminated screenings were added to the pelleted diets compared to the mash diets in our previous study, which was attributed to the lack of homogeneity of ergot contamination within the screenings. However, based on the results of the present study, pelleting may also have increased the apparent concentration of *R* epimers in our previous study. Less ergot added to the pelleted diets in our previous study is more likely to be responsible for the increased growth performance observed in lambs fed pelleted diets. The ergot used in previous studies was not evaluated in the current study due to it being stored for years. Significant shifts in epimers can occur in ergot alkaloids stored at 20 °C over a six-week period [19].

### 3.2. Ergot Alkaloid Analyses of Supplements and Complete Diets

Matrix effects influencing assay of ergot alkaloids have been previously reported, including pH [20], presence of solvents such as water [18], extraction of non-target compounds along with alkaloids [21], and likely others as yet to be determined influences. However, the magnitude of the difference in alkaloid concentrations depending on the matrix of the sample evaluated in the present study was surprising, with EM varying by more than 1 mg/kg in total alkaloids depending on whether a grain or silage-containing feed matrix was assayed. Although it would be tempting to blame a lack of homogeneity in the ergot-contaminated screenings for this variation, matrix effects likely also contributed to variation in alkaloid concentrations, as the supplement and complete diet differed in both pH and water content. In addition, methods were employed to increase the homogeneity of ergot alkaloids in diets, such as grinding ergot-contaminated screenings through a 1 mm screen, extensive mixing, and preparation of a single batch of supplement. Regardless of the causes of the variation in alkaloid analyses between samples of supplement and complete diet, they exacerbated the difficulty in predicting possible ergotism in cattle. Ergot alkaloid concentrations covered such a wide range (1.6 to 2.72 mg/kg) depending on the feed matrix analyzed, that they would be almost useless for evaluating potential risks to cattle.

### 3.3. Growth Performance of Cattle and Physiological Impacts

The concentrations of ergot alkaloids required to produce negative physiological effects, even in livestock of similar types and ages, have varied greatly among studies. Danicke and Diers [22] recognized the lack of usefulness of ergot alkaloid concentrations for a risk evaluation for livestock in general “and pigs in particular”, even without the matrix-related differences in alkaloid concentrations identified in the present study. In contrast to alkaloid concentrations, which are subject to matrix effects and lack of homogeneity, the concentration of the hormone prolactin has been a more reliable predictor of the physiological effects of ergot [5,11,23]. However, as concentrations of serum prolactin did not differ among treatment groups, the lack of impact of ergot treatments on growth performance is not surprising.

To determine if there were more subtle impacts of feeding ergot than could be seen from growth performance or feed intake, infrared thermography was used to measure the temperatures at three body locations. Ergot alkaloids are known to be potent vasoconstrictors [5,8], and extremities, such as the ears, feet, and tail, have developed gangrene and necrosis after exposure of cattle to ergot [24]. No difference in the temperatures of extremities was observed, even though measurements were made during winter months when vasoconstriction would be heightened due to cold. Perhaps with the covering hair layer, infrared thermography may lack sensitivity to detect mild vasoconstriction due to ergot exposure. Cowan et al. [17] detected decreased artery diameter using ultrasonography in beef cattle fed diets containing only 0.5 mg/kg alkaloids, approximately one-fifth to one-third of the alkaloid concentrations we used.

Although hair cortisol has been indicative of long-term stress in cattle [15], hair cortisol differed only among EP- and CP-fed cattle, likely for reasons other than exposure to ergot alkaloids, as the EM treatment was not similarly affected. The relationship between hair cortisol and exposure to ergot or other mycotoxins has not previously been studied, and additional studies will be needed to determine if such a relationship does exist. Similar to cortisol, haptoglobin was not affected by feeding ergot, but future studies are needed to determine whether higher concentrations of ergot alkaloids would negatively influence serum haptoglobin. The only physiological measures that demonstrated possible negative effects of exposure to ergot were CBC parameters, which, to our knowledge, have not been previously reported for cattle receiving diets containing cereal ergot. A recent study [25] reported an impact on CBC in cattle grazing fescue contaminated with ergot. The alkaloids present in fescue and cereal ergot differ [24], but similar to our results, Alfaro et al. [25] found reduced concentrations of hemoglobin and total white blood cells in cattle exposed to fescue ergot. In contrast to our results, these authors also found elevated red blood cell distribution width in cattle fed fescue ergot, which was attributed to iron-deficiency anemia [26]. Heat stress and reduced growth performance was also noted by Alfaro et al. [25], showing a closer relationship between CBC results and physiological effects of ergot than in the present study. Reduced white blood cells are indicative of reduced immune function [27], while reduced hemoglobin concentrations are signs of anemia [26]. However, as no other physiological effects of ergot were noted, and animal performance was not impacted, the changes in CBC values with feeding ergot in the present study are more likely a warning of potential problems from higher doses of alkaloids than being of immediate concern.

## 4. Conclusions

Anecdotal evidence from the feed industry has suggested that pelleting increases symptoms of ergotism in livestock. However, results from the present study demonstrated that pelleting did not enhance the negative physiological effects of ergot, although concentrations of ergot alkaloids detected in pelleted grain-based matrixes may be increased compared to mash feeds of similar composition. Possibly, the heat and pressure associated with pelleting may increase the ease of extraction of the ergot alkaloids during the assay. Accordingly, the 1 mg/kg limit proposed by the Canadian Food Inspection Agency for ergot alkaloids in the diets of cattle may unnecessarily penalize pelleted feed unless it is assayed in a lower-pH, higher-moisture matrix such as silage. Additionally, as total ergot alkaloids in diets between 1.6 and 2.7 mg/kg had no negative effects on cattle, other than suppressed CBC parameters, a 1 mg/kg limit for total alkaloids may be too restrictive for feeder calves. Ergot alkaloids are unstable during storage, and temperature and matrix effects make them difficult to measure. Improved assays are required before alkaloid concentrations can reliably predict risk to livestock or be used with any degree of confidence in regulatory applications.

## 5. Materials and Methods

### 5.1. Heating and Pelleting Ergot-Contaminated Screenings

Samples of ergot-contaminated grain were obtained from 15 locations in Alberta and Saskatchewan. An approximately 500-g subsample from each source was then transported to Romer labs in Tulln, Austria, and analyzed for concentrations of *R* and *S* forms of six alkaloids commonly associated with cereal ergot (ergocryptine, ergocornine, ergocristine, ergometrine, ergosine, and ergotamine) using HPLC-MS/MS [28]. Based on these results, five bulk totes with diverse alkaloid profiles were selected for the heating and pelleting studies (Table 1). Sub-samples (500 g) were spread in a single kernel layer on aluminum trays prior to heating for 10 min at 60, 80, 120 or 190 °C within a forced-air drying oven (Model OVF-400-80, Cole Parmer, Quebec, QC, Canada). To make pellets, 10 kg of the ergot-contaminated grain was first ground through a 1 mm screen in a hammermill (Eliminator E-1912-TF, Bliss Industries, Ponca City, OK, USA), and then mixed for 10 min in a Hobart M 802 mixer (Hobart Food Equipment Canada, Toronto, ON, Canada) with 80 kg of barley chop and 10 kg of canola meal before being pelleted at the Agriculture and Agri-Food Canada (AAFC) Research and Development Center Lethbridge, AB feed mill. Samples (500 g) of the heated and/or pelleted ergot were then sent to Romer labs for alkaloid analyses, as previously described.

### 5.2. Cattle and Feeding

Protocol #1905 was reviewed and approved on 15 March 2021, by the AAFC Animal Care Committee according to the guidelines of the Canadian Council on Animal Care [29], and the study was conducted between September and December, 2021. Forty-eight Angus cross steers, seven to eight months of age with an average live weight of 290 ± 4.4 kg, were used in the study. Cattle were transported to the AAFC Lethbridge feeding facility and placed in group pens to allow them to adapt to the facilities and standard high-forage backgrounding diets with no added ergot (Table 9) for 30 days (d). Steers were subsequently stratified by weight and randomly assigned to treatments and individual pens within the Individual Feeding Barn. Steers received feed and water on an ad libitum basis, and diets were delivered at 8:00 am each day after mixing silage with dry ingredients in an automated mixer wagon (Data Ranger, American Calan Inc., Northwood, NH, USA). Control diets (no added ergot) were fed before the ergot diets, and the Data Ranger tub was cleaned daily after feeding. Feed refusals from each steer were collected on a weekly basis, weighed, and sub-sampled, along with diets, with 600 g of each saved for further analysis.

### 5.3. Diet Preparation

Contaminated rye screens obtained from Enchant, Alberta (sample 20; Table 1) were selected as the source of ergot for the feeding study. Ergot was incorporated into the diets by substituting ergot-contaminated screenings for barley with the supplement. Other than the supplement, no other ergot was added to the diets. Complete diets consisted of barley grain, barley silage, canola meal, and supplements (Table 9). Dietary treatments included Ergot Mash (EM), Ergot Pelleted (EP), Control Mash (CM), and Control Pelleted (CP), based on the form of the supplement and the addition of ergot. Ergot was not subjected to heat treatments prior to pelleting or dietary inclusion. To maximize the homogeneity of the ergot alkaloids within the supplements, contaminated rye screenings were first mixed in a Hobart mixer for 20 min. A 31.7 kg aliquot of thoroughly mixed screenings was then processed through a hammermill, as described above, and mixed with other supplement ingredients for 20 min in a Sullivan Strong Scott SO mixer (Sullivan Strong Scott Ltd., Richmond, BC, USA). Half of the supplement was then pelleted, exposing the ergot to 90–100 °C and 4 bars pressure, with the remainder left in mash form. Control (no added ergot) supplements were also prepared in a single batch, with half pelleted under the same conditions as the added ergot treatment.

### 5.4. Cattle Growth Performance, Blood, and Hair Sampling, and Infra-Red Thermography

The cattle were weighed on d 0, 1, 22, 43, 63, and 64 using a hydraulic squeeze (Hi-Hog, Calgary, AB, Canada). Average daily gain (ADG) was determined by dividing weight gain (mean of d 63 and d 64 live weight–mean of d 0 and d 1 live weight) by the number of days in the study. Gain:Feed was calculated as the ratio between ADG and dry matter intake (DMI; kg of live weight gain/kg DMI). Blood and hair samples, infrared thermography measurements, and rectal temperatures were collected on d 1, 22, 43, and 64 for all cattle. Blood samples were drawn by jugular venipuncture using 10 mL non-additive tubes (BD vacutainer; Becton Dickinson Co., Franklin Lakes, NJ, USA) for haptoglobin and prolactin analysis, while another blood sample using 6 mL EDTA tubes (BD vacutainer) was collected for Complete Blood Count (CBC) analyses. Hair samples were clipped from the forehead as close to the skin as possible using an electric razor, as described by Moya et al. [15], for subsequent cortisol analyses. Temperature of the ears, tail, and coronary band region of the front legs were assessed using thermographic images produced by an FLIR i60 infrared camera (FLIR Systems Ltd., Burlington, ON, Canada) and analyzed using FLIR Tools version 6.4. Temperatures were recorded as an average of three points at each of three locations: along the center rib of the ear, on the tail below the tail head, and across the coronary band. Rectal temperature was measured with a digital thermometer (Sharptemp V digital thermometer; Cotran Corporation, Portsmouth, RI, USA).

### 5.5. Analyses of Blood and Hair

Blood collected in non-additive tubes remained at room temperature for two hours (h) before centrifugation at 2000× *g* for 15 min at 4 °C. Serum was then decanted and stored frozen at −40 °C until further analysis. Complete blood counts (CBC) were analyzed using a Hema True Hematology Analyzer (Heska, Loveland, CO, USA) as described by Melendez et al. [16]. Quantification of haptoglobin used a colorimetric assay (Tridelta Development Ltd., Maynooth, Co., Kildare, Ireland) following the manufacturer’s instructions. Intra- and inter-assay CVs were 6.3 and 10.4, respectively. For prolactin analyses, an ELISA Kit specific for bovine prolactin (CEA846Bo, Cloud-Clone Corp., Katy, TX, USA) was used with a limit of detection of 1.02 ng/mL. Intra- and inter-assay CVs were 4.2 and 11.5, respectively.

Hair samples were processed using a modified version of the methods described by Moya et al. [15]. Hair was washed with 5 mL of 70% ethanol and dried for 7 d at room temperature prior to ball grinding (MM 400, Retsch Inc., Newtown, PA, USA) in 10 mL stainless steel jars with 10 mm stainless steel balls. Ground hair (50 mg) was then added to 7 mL glass scintillation vials with 1.5 mL of methanol before shaking at 100 rpm for 16 h at 30 °C. After incubation, a commercial ELISA assay was used to determine cortisol concentrations (Salimetrics LLC, State Collage, PA, USA), as described by Moya et al. [15]. Intra- and inter-assay CVs were 5.9 and 12.7, respectively.

### 5.6. Feed Analysis

The pH of the complete diets and the ergot-containing supplements were analyzed using methods adapted from Bernardes et al. [30]. A 25 g aliquot was added to 100 mL of double distilled water, mixed for 5 min, and allowed to equilibrate for 10 min. The pH was then measured using an Accumet AB150 pH meter (Fisher Scientific, Ottawa, ON, Canada). Other analyses of diets and orts were performed by a commercial laboratory (Dairy One, Ithaca, NY, USA). Crude protein was determined by combustion of a 1 mm ground dry sample using a CN628 Carbon/Nitrogen Determinator (Leco Corp., St. Joseph, MI, USA) according to AOAC method 990.03 [31]. Acid detergent fiber (ADF) was determined using solutions described in AOAC 973.18. [31]. Samples of diets or orts (0.5 g) were added to the filter bags and digested for 75 min in 2 L of ADF solution in an Ankom Delta digestion unit (Ankom Technology, Macedon, NY, USA). For neutral detergent fiber (NDF), the methods of Van Soest et al. [32] were used in an Ankom Delta digestion unit. Samples (0.5 g) were added to the filter bags and digested for 75 min in 2 L of NDF solution, with 4 mL of α-amylase and 20 g of sodium sulfite added.

### 5.7. Statistical Analysis

For the initial heating and pelleting study, concentrations of *R* and *S* epimers were normalized by log transformation prior to analyses using the MIXED model of SAS (SAS Inst. Inc., Cary, NC, USA) with treatment (heat, pelleting, heat and pelleting) fixed effects and sample a random effect. For the performance study, the MIXED model was used in analyses of initial live weight, final live weight, average daily gain (ADG), and feed efficiency (dry matter intake (DMI)/kg liveweight gain) in a completely randomized design with ergot inclusion (control, no ergot vs. ergot), form (mash vs. pelleted), and ergot-by-form interaction as fixed effects and steer within each treatment as a random effect. Prolactin, cortisol, and haptoglobin data were normalized by log transformation prior to analyses. A repeated measures analysis was used for prolactin, haptoglobin, cortisol, infrared thermography, and dry matter intake analyses, with week as the repeated variable, steer within diet × treatment as a random effect, and day 1 measurements as a covariate. For these analyses, the treatment means were compared using the LSMEANS linear hypothesis test of SAS. As the CBC data were not normally distributed, distributions from the exponential family (gamma, inverse Gaussian, log-normal, exponential, and shifted t) were tested and selected for each parameter based on model fit statistics (Bayesian information criterion; BIC) within the GLIMMIX procedure of SAS. Covariance structures for the CBC analyses were also selected based on the BIC values. Significance for all analyses was declared at *p* ≤ 0.05.

## Figures and Tables

**Table 1:** Mean alkaloid concentrations (µg/kg, log_10_) and alkaloid ratios for grain screening sources from Alberta selected for heating and pelleting studies (*n* = 5).

Alkaloid	Sample Identification Number (Cereal Type)					
	5 (Rye)	7 (Rye)	20 (Rye)	23 (Barley)	24 (Rye)	SEM
ergocryptine	10.78 ^b^	9.88 ^a^	10.50 ^ab^	11.70 ^c^	11.68 ^c^	0.09
ergocryptinine	9.82 ^bc^	8.74 ^a^	9.26 ^ab^	10.15 ^c^	9.80 ^bc^	0.29
ergocornine	10.58 ^b^	9.67 ^a^	9.84 ^a^	11.05 ^c^	11.05 ^c^	0.16
ergocorninine	9.63 ^b^	8.94 ^a^	8.97 ^a^	10.19 ^c^	9.62 ^b^	0.14
ergocristine	12.98 ^b^	12.20 ^a^	12.47 ^a^	13.95 ^d^	13.40 ^c^	0.11
ergocristinine	11.65 ^d^	10.73 ^a^	11.03 ^b^	12.41 ^e^	11.31 ^c^	0.09
ergometrine	10.68 ^ab^	10.33 ^a^	10.88 ^ab^	11.99 ^c^	11.35 ^bc^	0.26
ergometrinine	9.17 ^de^	8.23 ^ab^	8.81 ^cd^	9.62 ^e^	8.60 ^bc^	0.18
ergosine	9.98 ^b^	9.19 ^a^	9.58 ^ab^	10.84 ^c^	10.68 ^c^	0.14
ergosinine	8.84 ^b^	8.09 ^a^	8.59 ^b^	9.70 ^c^	9.35 ^c^	0.15
ergotamine	11.80 ^bc^	11.04 ^a^	11.40 ^ab^	12.61 ^d^	12.22 ^cd^	0.16
ergotaminine	10.45 ^b^	9.58 ^a^	10.04 ^ab^	11.21 ^c^	10.45 ^b^	0.16
Total *R* epimers	13.53 ^c^	12.75 ^a^	13.09 ^b^	14.47 ^e^	14.03 ^d^	0.09
Total *S* epimers	12.27 ^c^	11.32 ^a^	11.68 ^b^	12.29 ^d^	12.08 ^c^	0.09
Total alkaloids	13.79 ^c^	12.98 ^a^	13.31 ^b^	14.68 ^e^	14.18 ^d^	0.08
Ratios of *R:S* epimers						
ergocryptine:ergocryptinine	0.96 ^a^	1.14 ^ab^	1.24 ^ab^	1.55 ^b^	1.88 ^bc^	0.16
ergocornine:ergocorninine	0.95 ^a^	0.72 ^a^	0.87 ^a^	0.86 ^a^	1.43 ^b^	0.09
ergocristine:ergocristinine	1.32 ^a^	1.47 ^a^	1.44 ^a^	1.53 ^a^	2.09 ^b^	0.11
ergometrine:ergometrinine	1.51 ^a^	2.10 ^b^	2.08 ^b^	2.36 ^bc^	2.74 ^c^	0.20
ergosine:ergosinine	1.14 ^b^	1.10 ^b^	0.99 ^a^	1.14 ^b^	1.34 ^c^	0.04
ergotamine:ergotaminine	1.35	1.46	1.36	1.40	1.77	0.24

^abcde^ Means within a row with different superscripts differ (*p* < 0.05).

**Table 2:** Effects of heating on alkaloid concentrations (µg/kg, log_10_) and alkaloid ratios for grain screening samples evaluated (*n* = 5).

Alkaloid	Heating					Significance		
	None	60 °C	80 °C	120 °C	190 °C	Linear	Quadr.	SEM
ergocryptine	10.52	10.81	10.90	10.85	10.93	0.005	0.02	0.02
ergocryptinine	9.01	9.11	9.18	9.16	9.63	0.01	0.05	0.04
ergocornine	10.05	10.43	10.51	10.48	10.51	0.03	0.06	0.05
ergocorninine	9.17	9.17	9.23	9.26	9.63	0.005	0.02	0.02
ergocristine	12.73	12.88	12.97	12.94	13.05	0.04	0.36	0.04
ergocristinine	10.91	10.89	10.97	10.96	11.50	0.006	0.01	0.03
ergometrine	11.42	11.48	11.53	11.55	11.47	0.13	0.06	0.02
ergometrinine	8.43	8.59	8.62	8.74	9.42	0.001	0.004	0.02
ergosine	9.87	9.99	10.04	10.02	10.06	0.10	0.51	0.05
ergosinine	8.58	8.56	8.71	8.71	9.10	0.01	0.05	0.04
ergotamine	11.63	11.68	11.77	11.73	11.79	0.09	0.55	0.03
ergotaminine	9.89	9.96	10.02	10.02	10.52	0.006	0.02	0.03
Total *R* epimers	13.36	13.48	13.57	13.54	13.61	0.03	0.20	0.03
Total *S* epimers	11.55	11.56	11.64	11.64	12.15	0.004	0.01	0.03
Total alkaloids	13.52	13.62	13.71	13.68	13.83	0.02	0.86	0.03
Ratios of *R:S* epimers								
ergocryptine:ergocryptinine	1.51	1.71	1.72	1.68	1.30	0.14	0.03	0.06
ergocornine:ergocorninine	0.88	1.26	1.28	1.22	0.89	0.72	0.006	0.03
ergocristine:ergocristinine	1.82	1.99	2.00	1.98	1.54	0.008	0.002	0.02
ergometrine:ergometrinine	2.99	2.89	2.90	2.82	2.05	0.002	0.007	0.01
ergosine:ergosinine	1.29	1.37	1.32	1.30	0.96	0.01	0.02	0.03
ergotamine:ergotaminine	1.75	1.72	1.75	1.71	1.27	0.002	0.003	0.01

**Table 3:** Effects of pelleting on alkaloid concentrations (µg/kg log_10_) and alkaloid ratios for grain screening samples evaluated (*n* = 5).

Alkaloid	Pelleting		
	Yes	No	SEM
ergocryptine	11.06	10.76	0.16
ergocryptinine	9.73	9.37	0.20
ergocornine	10.50	10.38	0.10
ergocorninine	9.55	9.39	0.10
ergocristine	13.10 ^b^	12.90 ^a^	0.07
ergocristinine	11.61 ^b^	11.24 ^a^	0.06
ergometrine	10.67 ^a^	11.41 ^b^	0.17
ergometrinine	8.81	8.96	0.12
ergosine	9.97	10.14	0.10
ergosinine	9.03	8.79	0.10
ergotamine	11.90	11.73	0.11
ergotaminine	10.52 ^b^	10.17 ^a^	0.11
Total *R* epimers	13.66 ^b^	13.49 ^a^	0.07
Total *S* epimers	12.24 ^b^	11.88 ^a^	0.06
Total alkaloids	13.89 ^b^	13.68 ^a^	0.06
Ratios of *R:S* epimers			
ergocryptine:ergocryptinine	1.32	1.39	0.11
ergocornine:ergocorninine	0.95	0.99	0.07
ergocristine:ergocristinine	1.49	1.65	0.07
ergometrine:ergometrinine	1.86 ^a^	2.46 ^b^	0.12
ergosine:ergosinine	1.11	1.18	0.03
ergotamine:ergotaminine	1.38	1.53	0.19

^ab^ Means within a row with different superscripts differ (*p* < 0.05).

**Table 4:** Effects of a combination of heating and pelleting heating on alkaloid concentrations (µg/kg log_10_) and alkaloid ratios for grain screening samples evaluated (*n* = 5). Treatments with the lowest (*p* < 0.05) alkaloid concentration or ratio in each row are highlighted in yellow.

Alkaloid	Heating and Pelleting Combination						Significance		
	Unheated Mash	Unheated Pellet	120 °C Mash	120 °C Pellet	190 °C Mash	190 °C Pellet	Treatment Combination	None Mash vs. Others	SEM
Ergocryptine	10.52	11.10	10.85	11.06	10.93	11.28	0.007	0.002	0.02
Ergocryptinine	9.01	9.35	9.16	9.73	9.63	10.13	0.01	0.02	0.04
Ergocornine	10.05	10.36	10.48	10.41	10.51	10.65	0.08	0.02	0.05
Ergocorninine	9.17	9.06	9.25	9.52	9.62	10.01	0.006	0.08	0.02
Ergocristine	12.73	13.19	12.94	12.90	13.05	13.20	0.07	0.03	0.05
Ergocristinine	10.91	11.15	10.96	11.53	11.50	12.15	0.005	0.03	0.03
Ergometrine	11.42	10.94	11.55	10.58	11.46	10.88	0.003	0.01	0.02
Ergometrinine	8.43	7.55	8.73	8.89	9.42	9.85	0.001	0.11	0.02
Ergosine	9.87	10.42	10.01	9.91	10.06	10.21	0.07	0.07	0.05
Ergosinine	8.58	8.77	8.71	9.01	9.10	9.45	0.02	0.05	0.04
Ergotamine	11.63	12.29	11.73	11.56	11.79	11.89	0.02	0.05	0.04
Ergotaminine	9.89	10.10	10.03	10.42	10.52	10.93	0.008	0.02	0.03
Total *R* epimers	13.36	13.80	13.54	13.50	13.61	13.71	0.04	0.02	0.03
Total *S* epimers	11.55	11.77	11.64	12.22	12.15	12.71	0.004	0.01	0.03
Total alkaloids	13.52	13.93	13.68	13.74	13.82	14.02	0.03	0.02	0.03
Ratios of *R:S* epimers									
ergocryptine:ergocryptinine	1.51	1.75	1.68	1.33	1.30	1.15	0.07	0.17	0.06
ergocornine:ergocorninine	0.88	1.30	1.22	0.88	0.89	0.64	0.02	0.01	0.03
ergocristine:ergocristinine	1.82	2.05	1.99	1.36	1.55	1.04	0.002	0.08	0.02
ergometrine:ergometrinine	2.99	3.38	2.81	1.68	2.04	1.02	0.002	0.002	0.01
ergosine:ergosinine	1.29	1.65	1.30	0.89	0.96	0.76	0.009	0.69	0.03
ergotamine:ergotaminine	1.75	2.19	1.70	1.13	1.27	0.96	0.001	0.07	0.01

**Table 5:** Mean ergot alkaloid concentrations and standard deviations in µg/kg unless otherwise labeled, and pH of complete diets and pelleted or mash ergot supplements from three samples collected monthly. Ergot was added only to the supplement. Alkaloid concentrations in supplements are expressed on a complete diet basis for ease of comparison (supplement = 2.8% of the complete diet).

Ergot Alkaloid	Total Diet			Supplement		
	Control Mash	Control Pellet	Ergot Mash	Ergot Pellet	Ergot Mash	Ergot Pellet
Ergocryptine	0 ± 0	0 ± 0	139 ± 17	101 ± 15	123 ± 28	155 ± 37
Ergocryptinine	0 ± 0	0 ± 0	102 ± 28	71 ± 9	32 ± 18	52 ± 3
Ergocornine	0 ± 0	0 ± 0	76 ± 17	55 ± 11	77 ± 7	92 ± 29
Ergocorninine	0 ± 0	0 ± 0	80 ± 15	61 ± 10	26 + 5	31 ± 5
Ergocristine	5 ± 1	0 ± 0	880 ± 174	531 ± 38	747 ± 221	1000 ± 271
Ergocristinine	4 ± 2	0 ± 0	609 ± 119	381 ± 144	150 ± 31	274 ± 60
Ergometrine	0 ± 0	0 ± 0	97 ± 29	58 ± 7	77 ± 9	98 ± 7
Ergometrinine	0 ± 0	0 ± 0	31 ± 12	22 ± 4	19 ± 4	19 ± 3
Ergosine	0 ± 0	0 ± 0	42 ± 8	34 ± 7	43 ± 6	56 ± 8
Ergosinine	0 ± 0	0 ± 0	30 ± 9	27 ± 4	13 ± 6	18 ± 4
Ergotamine	2 ± 1	0 ± 0	397 ± 124	258 ± 97	239 ± 68	344 ± 71
Ergotaminine	0 ± 0	0 ± 0	239 ± 97	149 ± 26	55 ± 3	81 ± 6
Total alkaloids (mg/kg)	0.01 ± 0	0.00 ± 0	2.72 ± 0.2	1.75 ± 0.2	1.60 ± 0.1	2.22 ± 0.2
Total *R* epimers (mg/kg)	0.01 ± 0	0.00 ± 0	1.63 ± 0.1	1.04 ± 0.1	1.31 ± 0.1	1.74 ± 0.2
Total *S* epimers (mg/kg)	0.00 ± 0	0.00 ± 0	1.09 ± 0.1	0.71 ± 0.1	0.30 ± 0.05	0.47 ± 0.1
pH	4.7 ± 0.2	4.7 ± 0.1	4.7 ± 0.2	4.7 ± 0.1	5.6 ± 0.2	5.6 ± 0.1

**Table 6:** Nutrient and other analyses of diet, supplements, or orts. Diets sampled once per period (*n* = 3). Orts were collected on a weekly basis and pooled for each steer per period. Statistical analyses compare differences among orts. Ergot, added ergot vs. control. Form, mash vs. pellet. E × F, interaction of ergot and form.

Substrate	Complete Diet				Orts				Significance			
	Control Mash	Control Pellet	Ergot Mash	Ergot Pellet	Control Mash	Control Pellet	Ergot Mash	Ergot Pellet	SEM	Ergot	Form	E × F
Dry matter (%)	54.9	54.4	54.2	55.0	54.3	54.8	54.5	54.7	0.1	0.38	0.60	0.20
Crude protein (%)	12.4	12.2	13.0	13.3	14.9	13.9	14.4	14.0	0.2	0.33	0.003	0.23
Ash (%)	7.0	5.5	5.6	6.0	15.1	9.8	10.5	9.2	0.9	0.01	0.001	0.04
Acid detergent fiber (%)	24.3	22.5	22.8	22.5	16.5	21.8	20.5	23.8	1.3	0.02	0.001	0.47
Neutral detergent fiber (%)	35.8	34.4	33.8	32.8	24.3	31.4	29.7	35.0	1.7	0.01	0.001	0.59

**Table 7:** Growth performance, rectal temperature, and infrared thermography of extremities of steers (*n* = 48) individually fed backgrounding diets with or without added ergot, with the supplement in either pelleted or mash form over a 64-d period. Ergot, added ergot vs. control. Form, mash vs. pellet. E × F, interaction of ergot and form.

Parameter	Diet				Significance			
	Control Mash	Control Pellet	Ergot Mash	Ergot Pellet	SEM	Ergot	Form	E × F
Shrunk weight kgs D0	289.4	290.2	290.4	289.1	9.1	0.99	0.98	0.91
Shrunk weight kgs D64	369.7	381.2	382.2	375.2	10.1	0.74	0.82	0.36
Gain kgs	80.3	91.0	91.8	86.1	NA ^z^			
Average daily gain (kg/d)	1.25	1.42	1.43	1.34	0.1	0.57	0.67	0.17
Average feed intake kg/d	7.5	7.8	7.9	7.4	0.2	0.94	0.62	0.07
Gain:feed	0.169	0.185	0.184	0.185	0.01	0.45	0.77	0.05
Rectal temperature °C	39.6	39.5	39.5	39.5	0.1	0.49	0.39	0.69
Mean temperature of mid-rib of ear °C	23.0	20.8	21.0	20.5	0.5	0.19	0.11	0.26
Mean temperature of coronary band above hoof °C	22.3	21.4	21.5	21.4	0.3	0.19	0.11	0.26
Mean temperature of tail head °C	20.9	20.7	20.5	20.8	0.5	0.20	0.11	0.26

^z^ NA, not applicable, calculated by difference.

**Table 8:** Complete blood counts, prolactin, haptoglobin, and cortisol analyses of steers (*n* = 48) individually fed backgrounding diets with or without added ergot with the supplement in either pelleted or mash form over a 64-d period. Ergot, added ergot vs. control. Form, mash vs. pellet. E × F, interaction of ergot and form.

Parameter	Diet				Significance			
	Control Mash	Control Pellet	Ergot Mash	Ergot Pellet	SEM	Ergot	Form	E × F
Granulocytes (%)	31.92	28.84	27.19	26.42	1.02	0.01	0.17	0.41
Monocytes (%)	7.75	7.18	6.83	6.69	0.21	0.01	0.10	0.31
Lymphocytes (%)	60.33	63.98	65.98	66.97	1.18	0.001	0.07	0.27
Total granulocytes 10^3^/µL	3.24	2.92	2.74	2.38	0.15	0.01	0.09	0.91
Total monocytes 10^3^/µL	0.84	0.76	0.73	0.65	0.03	0.01	0.10	0.92
Total lymphocytes 10^3^/µL	6.11	6.43	6.42	5.83	0.22	0.52	0.56	0.05
Total white blood cells 10^3^/µL	10.19	10.11	10.16	8.77	0.30	0.02	0.15	0.03
Platelet count 10^3^/µL	348.4	285.7	354.7	281.0	11.0	0.94	0.001	0.63
Total RBCs 10^6^/µL	7.72	7.92	8.64	8.55	0.37	0.04	0.88	0.69
Mean platelet volume fL	6.26	6.24	6.24	6.05	0.08	0.17	0.17	0.27
RBC distribution %	27.06	27.07	26.79	26.13	0.23	0.011	0.17	0.15
RBC volume fL	36.50	37.53	36.69	39.53	0.43	0.86	0.001	0.04
Hemoglobin concentration g/dL	34.94	34.71	34.75	33.98	0.13	0.001	0.09	0.05
Mean hemoglobin pg	12.76	12.99	12.51	13.41	0.12	0.47	0.01	0.001
Hematocrit %	32.71	32.78	32.23	34.91	0.56	0.14	0.02	0.02
Serum prolactin ng/mL	55.3	47.7	54.3	59.7	0.34	0.84	0.84	0.26
Serum haptoglobin mg/mL	0.10	0.10	0.11	0.11	0.02	0.53	0.94	0.95
Hair cortisol pg/mg	3.0	2.8	2.9	3.3	0.1	0.14	0.64	0.04

**Table 9:** Experimental diets used in the cattle-feeding study.

Ingredients, DM Basis (%)	Control	Added Ergot
**Total mixed diet**		
Barley silage	60	60
Barley grain	30	30
Canola meal	5	5
Supplement	5	5
Total	100	100
**Supplement**		
Barley chop	54.8	48.0
Calcium carbonate	25.0	25.0
Canola meal	10.0	10.0
Iodized salt	3.0	3.0
Molasses	2.5	2.5
Urea	2.0	2.0
Vitamin premix	1.0	1.0
Canola oil	1.0	1.0
Vitamin E	0.7	0.7
Rye ergot screenings	0.0	6.8
Total	100	100

## Data Availability

Not applicable.
